# Correction: Psychological impacts from COVID-19 among university students: Risk factors across seven states in the United States

**DOI:** 10.1371/journal.pone.0273938

**Published:** 2022-08-26

**Authors:** Matthew H. E. M. Browning, Lincoln R. Larson, Iryna Sharaievska, Alessandro Rigolon, Olivia McAnirlin, Lauren Mullenbach, Scott Cloutier, Tue M. Vu, Jennifer Thomsen, Nathan Reigner, Elizabeth Covelli Metcalf, Ashley D’Antonio, Marco Helbich, Gregory N. Bratman, Hector Olvera Alvarez

The following information is missing from the Funding statement: This study was based upon work supported in part by the National Science Foundation EPSCoR Cooperative Agreement OIA-1757351, and was made possible by all universities who participated in this study, in particular, the Department of Parks, Recreation and Tourism Management in the College of Behavioral, Social and Health Sciences at Clemson University and the Department of Parks, Recreation and Tourism Management in the College of Natural Resources at North Carolina State University.

The results should be adjusted to describe how older students were at higher risk of psychological impacts from the COVID-19 pandemic than younger students. These adjustments include changes in the Abstract, Results, Discussion and Conclusion.

The following describes where results and interpretations are corrected. Again, these are exclusively limited to our findings for older students, rather than younger students, being at higher risk of negative psychological impacts from the COVID-19 pandemic.

In the Results subsection of the Abstract, there is an error in the last sentence. The correct sentence is: Multivariate modeling (mixed-effects logistic regression) showed that being a woman, having fair/poor general health status, being at least 25 years old, spending 8 or more hours on screens daily, and knowing someone infected predicted higher levels of psychological impact when risk factors were considered simultaneously.

In the Risk Factors subsection of the Results, there is an error in the third and fourth sentences of the fifth paragraph. The correct sentence is: Students who were women, fair/poor general health, being at least 25 years old, reporting 8 or more hours of screen time, and who knew someone infected with COVID-19 were more likely to be in the high profile. Non-Hispanic Asian students were marginally more likely to be in the high impact profile, p = .059.

In the same subsection, there is an error in the first three sentences of the sixth paragraph. The correct sentences are: Sensitivity analyses with a subsample of respondents from the representative sample at North Carolina State University identified a similar set of predictors of psychological impact levels (Table A3 and S1). Gender, age, general health, and knowing someone infected remained significant predictors. In contrast, screen time was no longer significant, but Non-Hispanic Asian and social class were significant, p = .022 and .0038, respectively.

In the Key findings and interpretation of results subsection of the Discussion, there is an error in the first sentence of the fourth paragraph. The correct sentence is: In multivariate models controlling, being a woman, being older (at least 25 years old), having poor/fair general health, reporting more screen time, and knowing someone infected were statistically significant risk factors.

In the same subsection, the eighth paragraph is incorrect. The correct paragraph is: Our finding that older students were at greater risk than younger students was unexpected. Younger students (i.e., 18 to 24 years old, regardless of academic status) tend to be more worried about their future education and ability to pay for college education than older students [10]. Younger people also engage in social media more than older people during the pandemic [12,82]. Given the dominance of the COVID-19 pandemic in the news, it could have been expected that younger "always-on" students were exposed to greater amounts of risk-elevating messages, which could have led to anxiety and poor mental health [16,75]. Since our data suggest the opposite, further investigation into social media use and reactions among younger and older students is called upon to understand why older students were at greater risk of psychological impacts.

In the Recommendations for universities subsection of the Discussion, there is an error in the first two sentences of the fourth paragraph. The correct sentences are: Colleges and universities also have a moral obligation to boost their outreach to particularly vulnerable groups–that is, populations at risk of high levels of psychological impact from COVID-19 [14]. As documented in the impact profiles of our study, people at increased risk include women, older students, students with pre-existing health concerns, students spending at least one-third of their day (including time spent sleeping) on screens, and students with family or community members who are infected with COVID-19.

In the Conclusion section, there is an error in the first sentence. The correct sentence is: Our cross-sectional study found that being a woman, being of older age, experiencing poor/fair general health, spending extensive time on screens, and knowing someone infected with COVID-19 were risk factors for higher levels of psychological impact during the pandemic among college students in the United States.

A coding error made the results for age in Table 6 and S3 Table incorrect. Please see the complete, correct Table 6 and S3 Table below.

**Table 6:** Results of mixed-effects binary logistic regression modelling likelihood of risk factors predicting assignment to high COVID-19 psychological impact profile for students in seven United States universities (*N* = 2,140) ^a^.

	Log odds (95% CI)
Female	**2.018 (1.675, 2.431**^**)**^ ^***^
Age (18 to 24)	**0.728 (0.582, 0.910**^**)**^ ^**^
Race/Ethnicity	
Non-Hispanic White	*1*.*153* (0.*828*, 1.*604*)
Non-Hispanic Asian	**1.*469* (0.9*85*, *2*.*190*)** ^**^**^
Class (Self)	0.908 (0.802, 1.028)
General Reported Health	**0.619 (0.497, 0.772**^**)**^ ^***^
BMI	1.036 (0.91, 1.178)
Time Use (Last 24 Hours)	
Screen time	**1.216 (1.008, 1.466)** ^*^
Outdoor time	0.947 (0.834, 1.076)
Exercise	1.039 (0.93, 1.161)
Academic Status (Graduate)	0.946 (0.755, 1.186)
Knowing Someone Infected	**1.453 (1.173, 1.799**^**)**^ ^***^
Marginal R^2^ / Conditional R^2^ (%)	6.8 / 7.3
ICC	0.005

Note:

^^^*p* < .10,

^*^p < .05,

^**^p < .01,

^***^p < .001.

Predictors with *p* < .10 shown in bold. Due to identification with multiple races and missing values or other categories (i.e., mixed, prefer not to answer) for race/ethnicity and gender, 2,140 responses were available for multivariate analysis. These models were adjusted for random effects of institutional affiliation, ICC = Intra-Class Coefficient.

## Supporting information

S3 Table Results of binomial logistic regression modelling likelihood of risk factors predicting high versus low/moderate levels of COVID-19 psychological impact for students at North Carolina State University, where a representative sample was collected (*N* = 1,312). (DOCX)
