# Supplementary material for: Psychological impacts from COVID-19 among university students: Risk factors across seven states in the United States
Source: PLoS One. 2021 Jan 7;16(1):e0245327. doi: 10.1371/journal.pone.0245327 (PMC7790395; doi:10.1371/journal.pone.0245327)
Supplement: S1 Fig — (DOCX) [file pone.0245327.s001.docx]

**S1 Fig.** Distributions and relationships between COVID-19 psychological impact survey items, including histograms, Pearson correlation coefficients, and scatter plots.

**
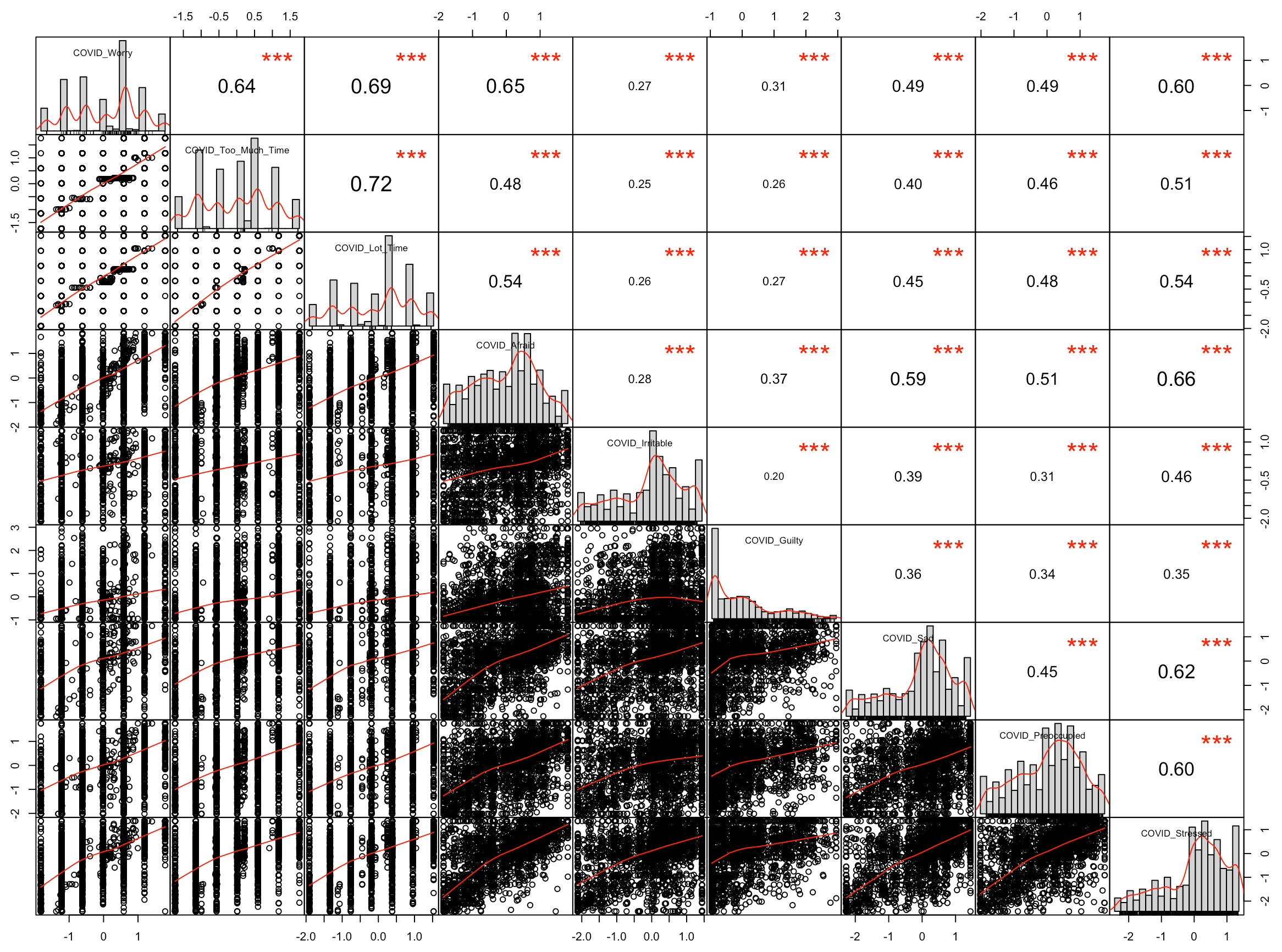
**
