# Supplementary figures and images for: Psychological impacts from COVID-19 among university students: Risk factors across seven states in the United States
Source: PLoS One. 2021 Jan 7;16(1):e0245327. doi: 10.1371/journal.pone.0245327 (PMC7790395; doi:10.1371/journal.pone.0245327)

**S2 Fig.** Diagram of EFA on COVID-19 psychological impact survey items.


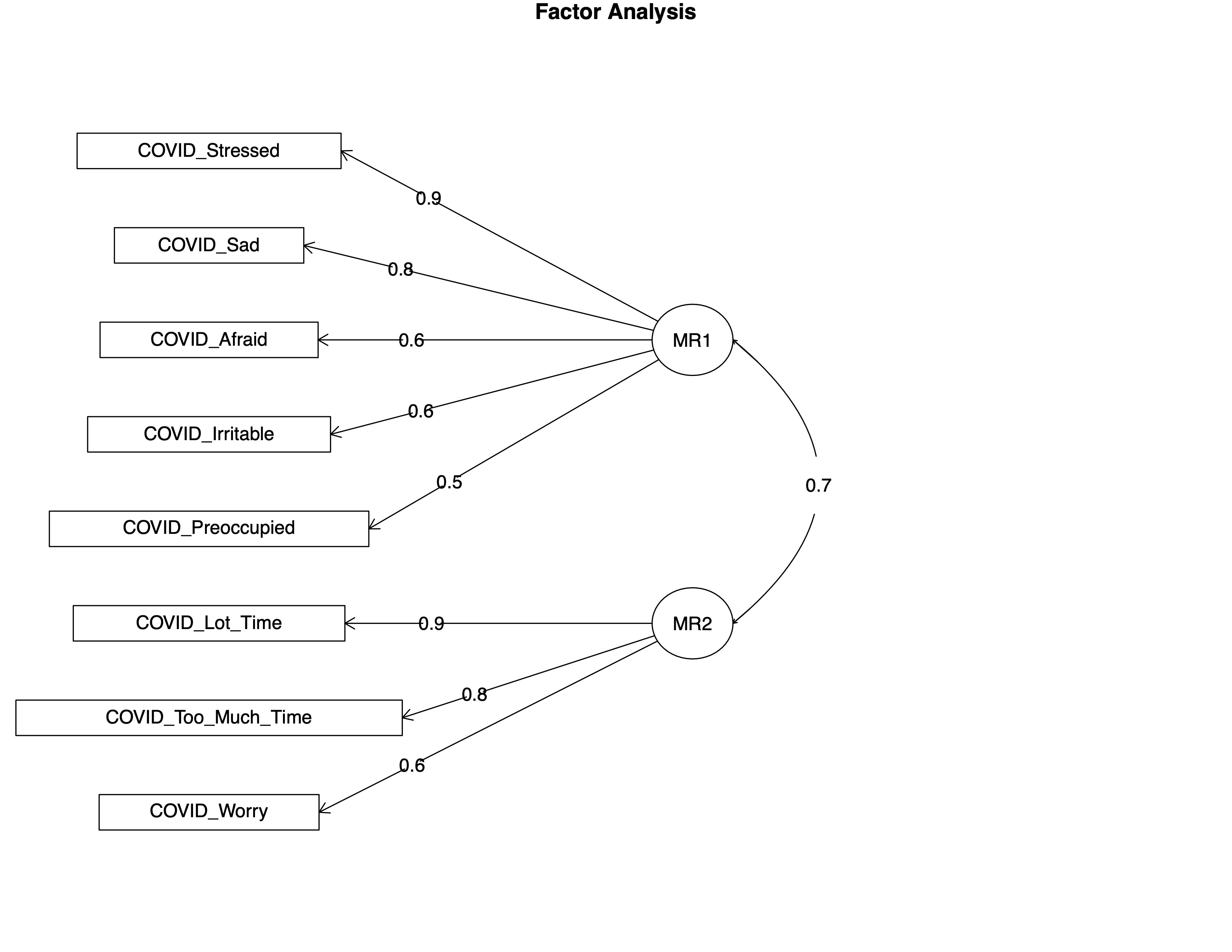

Supplement: S2 Fig — (DOCX) [file pone.0245327.s002.docx]

**S3 Fig.** Scree plot of EFA on COVID-19 psychological impact survey items.


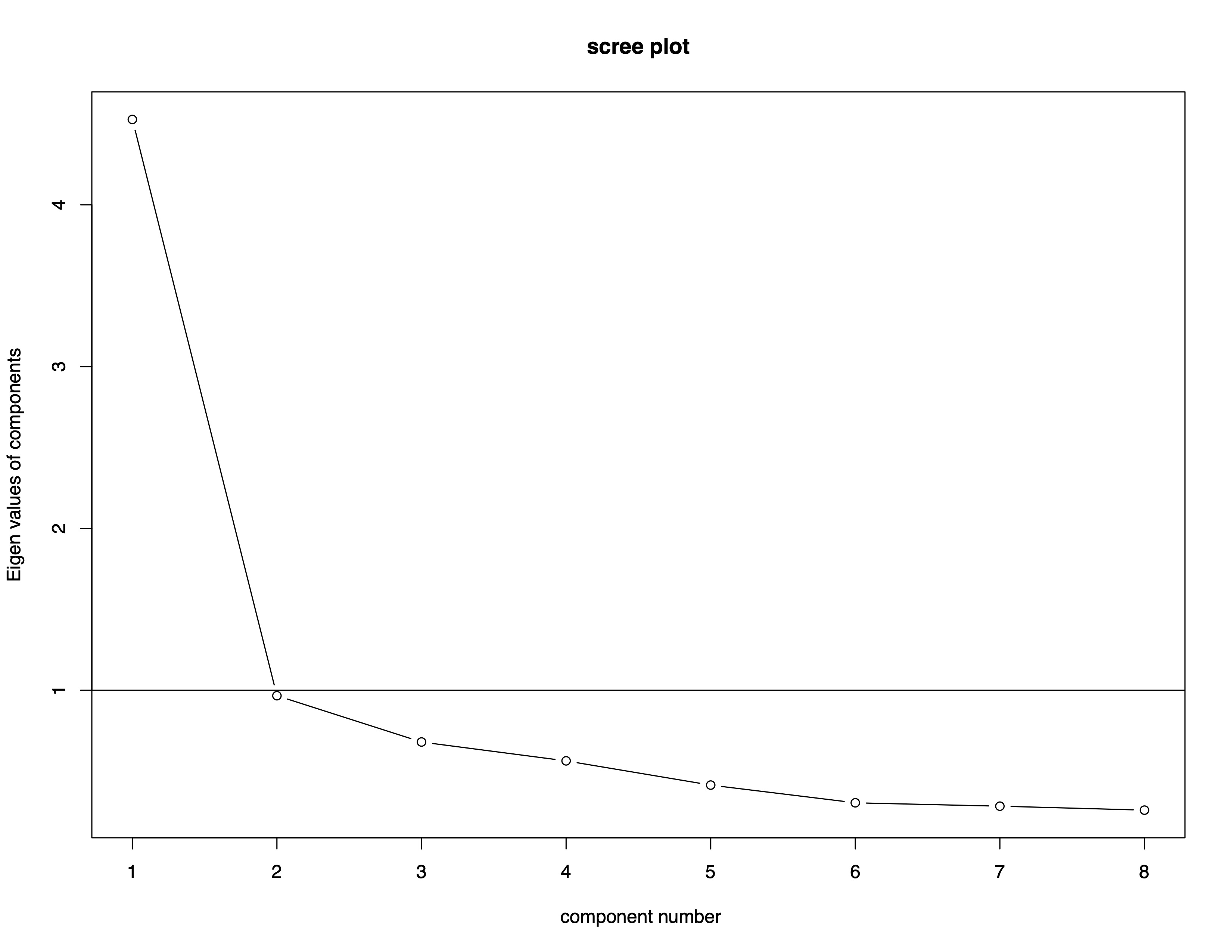

Supplement: S3 Fig — (DOCX) [file pone.0245327.s003.docx]

**S4 Fig.** Elbow plot of the information criteria for the latent profile analysis.


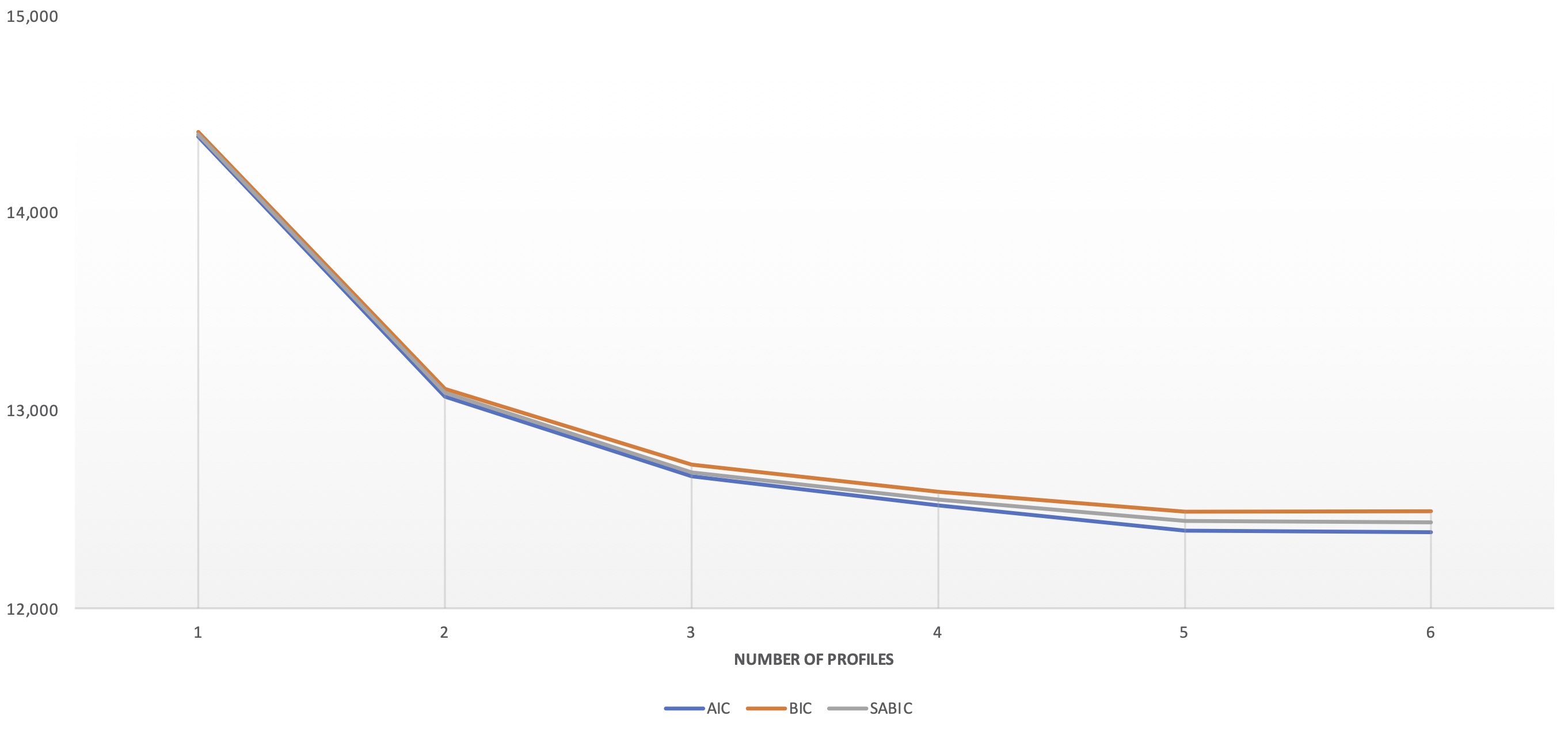

Supplement: S4 Fig — (DOCX) [file pone.0245327.s004.docx]
