# Supplementary material for: Psychological impacts from COVID-19 among university students: Risk factors across seven states in the United States
Source: PLoS One. 2021 Jan 7;16(1):e0245327. doi: 10.1371/journal.pone.0245327 (PMC7790395; doi:10.1371/journal.pone.0245327)

**S5 Fig.** Correlations between socio-economic measures and the two psychological impact profiles.


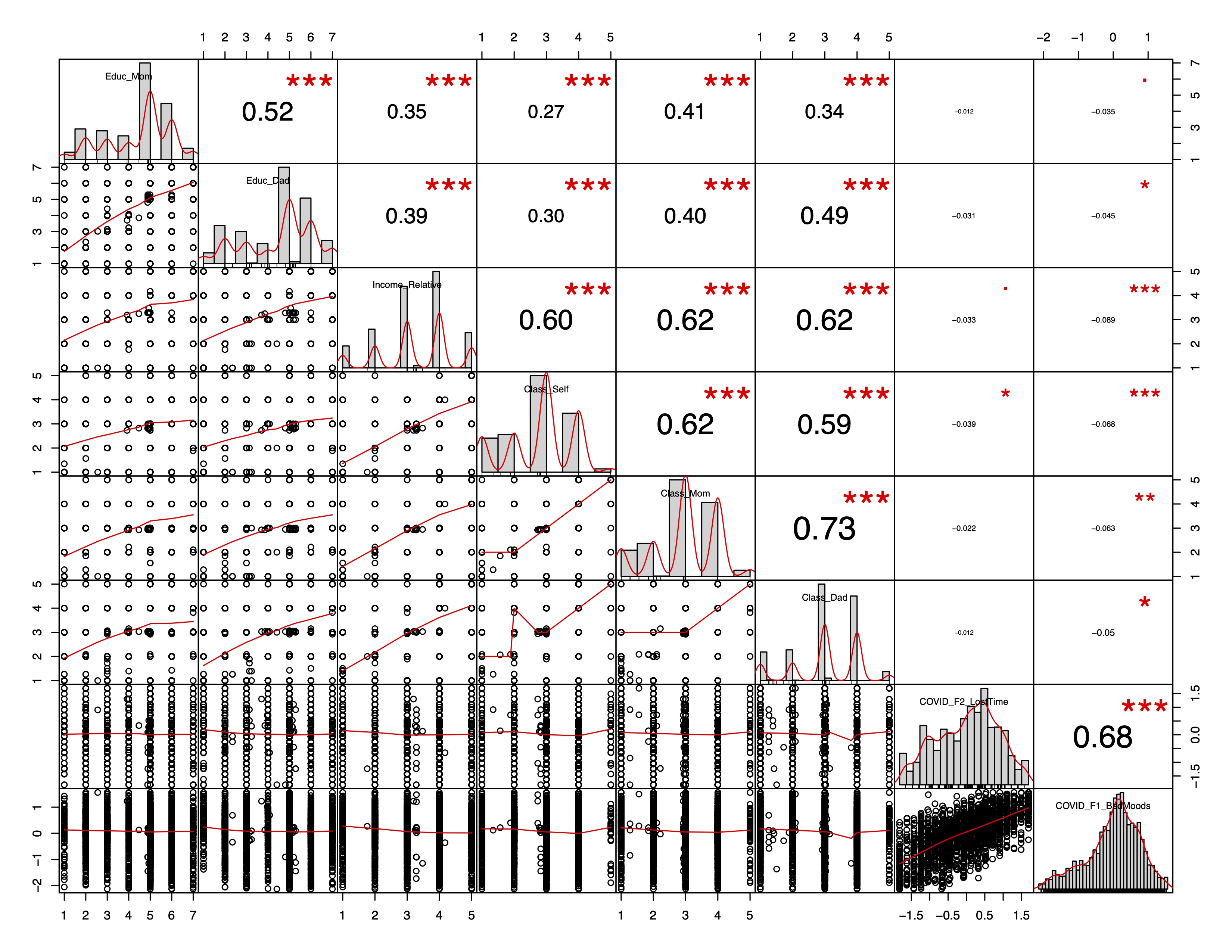

Supplement: S5 Fig — (DOCX) [file pone.0245327.s005.docx]
