# Supplementary material for: Psychological impacts from COVID-19 among university students: Risk factors across seven states in the United States
Source: PLoS One. 2021 Jan 7;16(1):e0245327. doi: 10.1371/journal.pone.0245327 (PMC7790395; doi:10.1371/journal.pone.0245327)

**S6 Fig.** Conditional mean values ("condval") and standard deviations of institutional affiliation (university) random effects from mixed-effects logistic regression predicting high versus medium/low psychological impact profile from COVID-19.


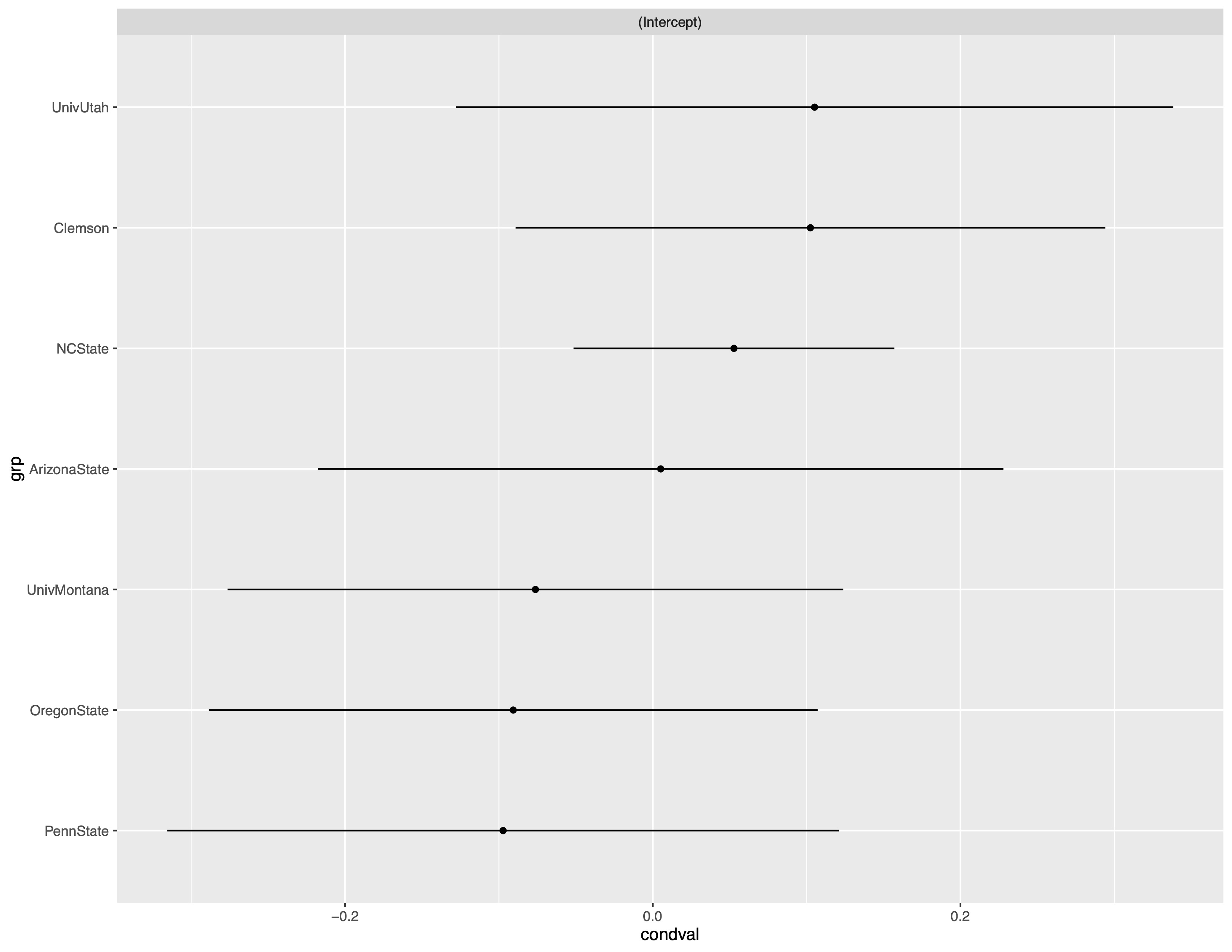

Supplement: S6 Fig — (DOCX) [file pone.0245327.s006.docx]
