# Supplementary material for: Psychological impacts from COVID-19 among university students: Risk factors across seven states in the United States
Source: PLoS One. 2021 Jan 7;16(1):e0245327. doi: 10.1371/journal.pone.0245327 (PMC7790395; doi:10.1371/journal.pone.0245327)
Supplement: S1 Table — (DOCX) [file pone.0245327.s007.docx]

**S1 Table.** Item loadings and fit statistics of EFA on COVID-19 psychological impact survey items.

| **Item** | **Factor 1** ("Emotional Distress") |  | **Factor 2** ("Worry Time") |
| --- | --- | --- | --- |
| Worry |  |  | 0.62 |
| Too Much Time |  |  | 0.81 |
| Lot of Time |  |  | 0.90 |
| Afraid | 0.59 |  |  |
| Irritable | 0.57 |  |  |
| Sad | 0.76 |  |  |
| Preoccupied | 0.51 |  |  |
| Stressed | 0.89 |  |  |
| Sum of the square factor loadings | 2.14 |  | 2.55 |
| Proportion variance explained | 0.27 |  | 0.32 |
| Cumulative variance explained | 0.46 |  | 0.32 |

*Note.* Factor loadings <.3 not shown.
