# Supplementary material for: Psychological impacts from COVID-19 among university students: Risk factors across seven states in the United States
Source: PLoS One. 2021 Jan 7;16(1):e0245327. doi: 10.1371/journal.pone.0245327 (PMC7790395; doi:10.1371/journal.pone.0245327)
Supplement: S2 Table — (DOCX) [file pone.0245327.s008.docx]

**S2 Table.** Fit indices, entropy and model comparisons for estimated latent profile analyses models.

| **Model** | **Log-Likelihood** | **AIC** | **BIC** | **SABIC** | **P-value of BLRT** | **Entropy** |
| --- | --- | --- | --- | --- | --- | --- |
| 1 | -7190.00 | 14388 | 14412 | 14399 | <.001 | 1 |
| 2 | -6527.00 | 13069 | 13110 | 13088 | <.001 | 0.756 |
| 3 | -6323.00 | 12666 | 12724 | 12693 | <.001 | 0.727 |
| 4 | -6246.00 | 12518 | 12594 | 12552 | <.001 | 0.684 |
| 5 | -6182.00 | 12396 | 12489 | 12438 | <.001 | 0.754 |
| 6 | -6173.00 | 12385 | 12496 | 12435 | <.001 | 0.664 |

*Note.* AIC = Akaike Information Criterion; BIC = Bayesian Information Criterion; SABIC = Sample-size Adjusted BIC; BLRT = Bootstrap Likelihood Ratio Test
