# Supplementary material for: Psychological impacts from COVID-19 among university students: Risk factors across seven states in the United States
Source: PLoS One. 2021 Jan 7;16(1):e0245327. doi: 10.1371/journal.pone.0245327 (PMC7790395; doi:10.1371/journal.pone.0245327)
Supplement: S3 Table — (DOCX) [file pone.0245327.s009.docx]

**S3 Table.** Results of binomial logistic regression modelling likelihood of risk factors predicting high versus low/moderate levels of COVID-19 psychological impact for students at North Carolina State University, where a representative sample was collected (*N* = 1,312).

|  | Log odds (95% CI) |
| --- | --- |
| Female | **0.64761 (0.4079, 0.8873)^***^** |
| Age (18 to 25) | **0.31381 (0.0243, 0.6033)^*^** |
| *Race/Ethnicity* |  |
| Non-Hispanic White | -0.23413 (-0.6267, 0.1584) |
| Non-Hispanic Asian | **0.30445 (-0.0244, 0.6333)^^^** |
| Class (Self) | **-0.23038 (-0.3865, -0.0742)^**^** |
| General Health | **-0.47023 (-0.7447, -0.1958)^***^** |
| BMI | 0.00569 (-0.1548, 0.1662) |
| *Time Use (Last 24 Hours)* |  |
| Screen time | 0.12501 (-0.1108, 0.3608) |
| Outdoor time | -0.03499 (-0.1944, 0.1244) |
| Exercise | -0.0108 (-0.1475, 0.1259) |
| Student Standing (Graduate) | -0.00383 (-0.2885, 0.2808) |
| Knowing Someone Infected | **0.36861 (0.0944, 0.6428)^**^** |
| Marginal R^2^ / Conditional R^2^ (%) | 6.8 / 6.8 |
| Log Likelihood (df) | -868 (13) |

*Note:* ^^^*p* < .10, ^*^*p* < .05, ^**^*p* < .01, ^***^*p* < .001. Predictors with *p* < .10 shown in bold.
