# Supplementary material for: Correction: Psychological impacts from COVID-19 among university students: Risk factors across seven states in the United States
Source: PLoS One. 2022 Aug 26;17(8):e0273938. doi: 10.1371/journal.pone.0273938 (PMC9417008; doi:10.1371/journal.pone.0273938)
Supplement: S3 Table — (DOCX) [file pone.0273938.s001.docx]

**S3 Table.** Results of binomial logistic regression modelling likelihood of risk factors predicting high versus low/moderate levels of COVID-19 psychological impact for students at North Carolina State University, where a representative sample was collected (*N* = 1,312).

|  | Log odds (95% CI) |
| --- | --- |
| Female | **0.648 (0.408, 0.887)^***^** |
| Age (18 to 25) | **-0.314 (-0.603, -0.024)^*^** |
| Race/Ethnicity |  |
| Non-Hispanic White | 0.234 (-0.158, 0.627) |
| Non-Hispanic Asian | **0.539 (0.0776, 1.000)^*^** |
| Class (Self) | **-0.230 (-0.386, -0.0742)^**^** |
| General Health | **-0.470 (-0.745, -0.196)^***^** |
| BMI | 0.00569 (-0.155, 0.166) |
| Time Use (Last 24 Hours) |  |
| Screen time | 0.125 (-0.111, 0.361) |
| Outdoor time | -0.0350 (-0.194, 0.124) |
| Exercise | -0.0108 (-0.148, 0.126) |
| Student Standing (Graduate) | -0.00383 (-0.289, 0.281) |
| Knowing Someone Infected | **0.369 (0.0944, 0.643)^**^** |
| Marginal R^2^ / Conditional R^2^ (%) | 6.8 / 6.8 |
| Log Likelihood (df) | -868 (13) |

Note: ^*^*p* < .05, ^**^*p* < .01, ^***^*p* < .001. Predictors with *p* < .10 shown in bold
